# Cobenefits of Replacing Car Trips with Alternative Transportation: A Review of Evidence and Methodological Issues

**DOI:** 10.1155/2013/797312

**Published:** 2013-07-16

**Authors:** Ting Xia, Ying Zhang, Shona Crabb, Pushan Shah

**Affiliations:** ^1^School of Population Health, The University of Adelaide, North Terrace, Adelaide, SA 5000, Australia; ^2^School of Public Health, The University of Sydney, Fisher Road, Sydney, NSW 2008, Australia; ^3^Environment Protection Authority, GPO Box 2607, Adelaide, SA 5001, Australia

## Abstract

It has been reported that motor vehicle emissions contribute nearly a quarter of world energy-related greenhouse gases and cause nonnegligible air pollution primarily in urban areas. Reducing car use and increasing ecofriendly alternative transport, such as public and active transport, are efficient approaches to mitigate harmful environmental impacts caused by a large amount of vehicle use. Besides the environmental benefits of promoting alternative transport, it can also induce other health and economic benefits. At present, a number of studies have been conducted to evaluate cobenefits from greenhouse gas mitigation policies. However, relatively few have focused specifically on the transport sector. A comprehensive understanding of the multiple benefits of alternative transport could assist with policy making in the areas of transport, health, and environment. However, there is no straightforward method which could estimate cobenefits effect at one time. In this paper, the links between vehicle emissions and air quality, as well as the health and economic benefits from alternative transport use, are considered, and methodological issues relating to the modelling of these cobenefits are discussed.

## 1. Introduction

Over the last century, the number of motor vehicles built, purchased, and used on roads globally has dramatically increased to meet people's travel demands. Although alternative fuels have been developed, more than 95% motor vehicles are still dependent on fossil fuels, a dependency which does not seem to be abating [1, 2]. Because of the large consumption of fossil fuels, transportation is regarded as a major contributor of greenhouse gases (GHGs). According to research conducted by Kahn Ribeiro and colleagues [3], a quarter of world energy-related GHG emissions can be attributed to transportation and nearly 85% of transportation-related GHG is exhausted by land transportation. Furthermore, it is predicted that transport energy usage will continue to increase at a rate of about 2% per year worldwide, whilst total transport energy usage and carbon emissions will be 80% higher than their current levels by 2030 [3]. 

It is widely acknowledged that exhaust fumes from motor vehicles contain a variety of air pollutants such as nitrogen dioxide (NO_2_), volatile organic compounds (VOCs), carbon monoxide (CO), and particulate matter (PM). Although the contribution of road transport to local pollution may vary depending on distinct local features, such as geographic and climatic features, the technology distribution of the national fleet, driving patterns and density [4], and vehicle emission is no doubt a significant source of air pollution, especially in highly car-dependent cites. The European Topic Centre on Air and Climate Change 2005 data [5] demonstrate that road transport accounts for about 42% of total NO_*x*_ (NO and NO_2_), 47% of total CO, and 18.4% of total PM emissions at European Union of 15 member states. 

To reduce the emissions from motor vehicles, mitigation strategies have been implemented in various countries. These mitigation strategies could be summarised as falling into three main approaches: (1) renovation of new vehicle technology, such as developing new energy sources for motor vehicles and elevating standards for emissions [6, 7]; (2) improvement of land use and urban planning, such as an establishment of bus rapid transit systems [8]; (3) travel behaviour change promotion in terms of promoting sustainable alternative transport use, such as public transport and active transport (e.g., cycling and walking), which has been a common approach in some European cities [9]. Besides the direct-core environmental benefit, the mitigation strategies of reshaping transport patterns via promoting mass transit and active transport have been increasingly recognised as an opportunity to gain great cobenefits. The definition of a cobenefit is “an additional benefit arising from an action that is undertaken for a different principal purpose” [10]. For example, both public transport and active transport will result in less dependency on fossil fuels and a reduction in traffic congestion. As a result of restricting vehicle use, air quality could be significantly improved and the health issues caused by air pollution could be alleviated. Additionally, active transport, in particular, also provides health benefit through regular physical activities. Moreover, economic improvements could also be gained from reduction of car use.

Exploring and understanding these cobenefits might provide invaluable information to policy makers in transport and land planning. However, to date, little research has been conducted in these areas. In order to improve understanding of the advantages of alternative transport, this paper aims to review, in detail, (1) the evidence regarding the health and economic cobenefits of alternatives to car travel, and (2) the methodological issues faced in previous studies in this field. Recommendations for further research are then discussed. 

## 2. Method

A literature search for reviewing papers published in English between 2002 to March 2013 was conducted using the main research databases PubMed, Scopus, Web of Science, and Google scholar along with searching of references on relevant organisations' websites including World Health Organization, the International Panel on Climate Change (IPCC), and relevant transport department websites. The search focussed on two purposes: first, a review of the broad literature relevant to effects of vehicle emissions in order to summarise benefits of alternatives to car travel. The search was conducted using a combination of keywords as follows: land/road transportation, vehicle emission, transport/traffic emission, air pollution, air quality, car trips, alternative transport, public transport, active transport, bicycling, cycling, and walking. The second purpose of focus was “co-benefits” studies, specifically in the transport sector. The major goal at this stage was to identify specific studies which conduct multiple benefits evaluation of alternative transport scenarios. These “co-benefits” papers were reviewed for specific issues within the methodology of modelling cobenefit effects from alternative transport scenarios. Review of methodological issues in the “co-benefits” studies were identified according to the following criteria: (i) whether the studies focused on transport sector; (ii) whether multiple benefits of alternative transport scenarios were evaluated, and (iii) whether projective models were used. Exclusion criteria were applied: first, those focused on the whole energy system rather than transport system; second, studies only evaluating single benefit of alternative transport scenarios and review papers. Except for unavailable papers, five “co-benefits” studies were identified and are listed in Table 1, with a summary of the scenario design, target populations, modelling method/tools, environmental and health indicators, and main findings.

### 2.1. Public Transport

Public transport, such as bus and train, is extensively used as a dominant travel mode in developing countries. Compared to private car, public transport has a larger carrying capacity. Trams, trains, and subways rarely get stuck in traffic congestion, and bus schedule can be flexibly arranged, with multiple buses able to travel the same route simultaneously, in response to peak times or to cater for special events [16, 17]. Although public transport is not defined as a “zero-pollutant” travel mode, its average emissions per passenger are far lower than that from cars. Furthermore, cleaner and more fuel efficient public transport is becoming more common in many countries, supporting a further reduction of GHG emissions and air pollution. For example, Compressed Natural Gas (CNG) buses have been used in several countries, such as USA, Brazil, Argentina, Italy, Pakistan, and New Zealand, for many years [18]. Additionally, walking to and from public transportation may help physically inactive populations achieve the recommended level of daily physical activity. A US study found that 29% of people who use public transit could achieve ≥30 minutes of physical activity a day solely by walking to and from transit [19]. A systemic review conducted by Rissel et al. [20] reported that public transport usage could increase physical activity per day by a range of 8–33 minutes. Public transport may not be attractive for local residents as private cars because it is less flexible and can take longer to travel to one's destination. Therefore, buses or trams are often not considered as a real alternative to cars. However, these problems could be counteracted by creating priority systems for public transport for traffic lights and building quality bus corridors or priority routes, which have been implemented in many countries such Korea, USA, and Australia [21–24]. Despite such government initiatives, public transport trips continue to account for only a small portion of total trips in many urban areas. In London and Sydney, for example, only about 10% of all trips are made by public transport, while over 70% of all trips are made by car [25, 26].

### 2.2. Active Transport

Active transport is another attractive environmentally friendly transport alternative, particularly for short journeys. Active transportation, including travelling on foot and by bicycle and other nonmotorised transport, is recognised as largely “zero-pollutant,” with respect to emissions of the travel itself (emissions are produced in the building, distributing, and servicing of bicycles, e.g.). The other advantage of the active transportation is flexible (or nonexistent) parking considerations and lower cost. At the moment, a large proportion of the total trips in most European cities are shorter than 2.5 km which is a distance relatively easy to be replaced by active transport: 44% in the Netherlands, 37% in Denmark, 41% in Germany, and 30% in UK [27]. Short trips also occupy a considerable percentage of total travel trips in major Australian cities. Taking Sydney as an instance, 20% of all trips made on an average weekday are less than 1 km, 35% are less than 2 km, and 60% are less than 5 km, which has been considered to be a suitable distant for walking or cycling [26]. However, the number of trips made by walking and cycling has declined significantly over the last 20 years [28]. It has been reported that cycling only occupies less than 3% of the total travel trips in some cities in UK, USA, and Australia [26, 27, 29]. This decline in cycling strongly reflects a high reliance on motor cars in modern society.

Despite the significant decline in the number of trips made by active transport, government efforts can play a considerable role in active transport promotion. It is estimated that a 52% increase in bicycle trips could be achieved in Australia by 2016, and a 71% rise by 2026 under a collaboration among the Australian Local Government Associations [30]. Given growing environmental concerns, many developed countries have conducted cycling promotion programmes to encourage active transportation. Countries like the Netherlands, Denmark, and Germany have been very successful in this endeavour. From 1950 to 1975, the percentage of trips using cycling decreased significantly by two-thirds in the Netherlands (from 50% to 85% of trips in 1950 to only 14–35% of trips in 1975) and Germany (fell by 78% from 1950 to 1975), as car ownership surged and cities started spreading out [27]. However, during that 25-year period, the governments of these countries focused on improving their cycling infrastructure, whilst imposing restrictions on car use; subsequently, the cycling share of trips increased by 25%. Currently, over 30% of trips to work or school are made by bicycle in the Netherlands and Denmark, whilst this percentage in Germany is 28% [27].

## 3. Evidence of Potential Benefits of Promoting Alternative Transport

### 3.1. Environmental Benefits

More than half of the world's population live in urban areas, and it has been estimated that the global urbanised population will reach five billion by 2030 [31]. Accordingly, air quality will be significantly affected due to increasing travel demands and related motor vehicle usage. However, air quality could be largely improved by implementing appropriate traffic controlling strategies especially in urban areas. During the 2008 Beijing Olympic Games [32], for instance, most Beijing residents chose public transit or cycling as their dominant mode of transport because a large portion of private and business cars were restricted in use according to Olympic traffic management. Consequently, a noteworthy reduction of traffic flow was noticed during the Olympic traffic control days and on-road air quality improved significantly: the average reduction rates of PM_10_, CO, NO_2_, and O_3_ reached 28%, 19.3%, 12.3%, and 25.2%, respectively [32]. Similarly, a three-month traffic restriction implemented during the Sino-African Summit was a remarkable success in air pollution control, reducing 40% NO_*x*_ emissions in Beijing [33]. Throughout the period of the 1996 Atlanta Olympic Games, as a result of traffic restrictions, peak daily ambient ozone concentrations dropped by 30% from the baseline measure, which led to a significant decrease of asthma cases [31]. Therefore, reducing motor vehicle usage can improve air quality with immediate short-term effect.

### 3.2. Health Benefits

#### 3.2.1. Health Benefit from Mitigation of Vehicle Emission Reduction

On one hand, transportation has been identified as being partly responsible for GHG effects, given that the emissions from motor vehicles contain large amounts of CO_2_, NO_*x*_, and CH_4_. Furthermore, it has been proven that GHG effect is the main cause of global warming. Cross-sectional studies conducted in different regions have shown that thousands of excess deaths could be caused with the increased frequency and intensity of extreme weather [34–36]. On the other hand, according to a recent WHO report, approximately 1.3 million premature deaths worldwide are attributed to outdoor air pollution in 2009 [37]. Recently, health impacts of vehicular air pollution have also attracted more and more public attention and academic research, with an increasing number of studies investigating the association between proximity to roads and population health. They reported that pollutant concentrations are higher in areas closer to motorways and decline gradually with distance from motorways [38, 39] and increasing mortality and morbidity have been observed in populations living near major roads [40–42]. Particularly, a Dutch cohort study enrolling 12,852 subjects with a 10-year followup illustrated that traffic intensity on the nearest road would increase mortality of natural causes, cardiovascular, respiratory, and lung cancer by 5%, 4%, 22%, and 3%, respectively [43]. Similarly, people living close to major roadways have an increased risk of coronary mortality [44]. In contrast, the risk has been found to decrease gradually when people move away from major roadways.

Transport-specific behavioural change programmes, including increasing mass transit use and active travel, are essential in relieving these adverse health effects. Woodcock and colleagues [11] evaluated the environmental and health benefits of various alternative transport scenarios by 2030 in London, UK, and Delhi, India. Their research indicated that about 122,000 premature deaths and thousands of disability-adjusted life-years (DALYs) caused by air pollution could be saved under alternative transport scenarios by 2030. Another study conducted in Mexico City evaluated five control options for the Program to Improve Air Quality in the Valley of Mexico: taxi fleet renovation, metro expansion, hybrid buses, liquefied petroleum gas (LPG), and cogeneration. The results showed that these five measures together could reduce approximately 1% PM10 exposure, 3% maximum ozone exposure, and more than 1.5 Mton (Metric Ton) CO_2_ equivalent emissions. Additional to the environmental benefits, these measures could also save nearly 100 lives and reduce 700 cases of chronic bronchitis each year.

#### 3.2.2. Health Benefit from Active Transport

Another potential health cobenefit comes from increased physical activity associated with active transport. According to Global Recommendations [45], adults aged 18–64 should do at least 150 minutes of moderate intensity aerobic activity per week. A person who walks or cycles 150 minutes a week or 30 minutes per week day could be grouped in the population conducting regular physical exercise on the basis of the World Health Organization (WHO) recommendation. Although such guidelines for physical activity have been provided for a long time, sedentary lifestyles still remain a global public health problem. To date, physical inactivity has been regarded as one of the most risky behavioural factors contributing to disease burden, especially in developed countries [46].

*Active Transport, Physical Activity, and Health Benefits Overall*. Active transport such as walking and cycling provides an opportunity to incorporate frequent physical activity into daily living, which could help people achieve recommended levels of physical activity. Moreover, various evidence of a positive association between active transport and health outcomes has been published [20, 47–51]. For example, a recent systematic review [52] summarised evidence of the health benefits of cycling and reported a strong inverse relationship between commuter cycling and all-cause mortality, cardiorespiratory fitness, cancer mortality and morbidity, and a clear positive dose-response relationship between the amount of cycling and body fitness, and incidence of overweight and obesity decrease.

A systematic review conducted by Woodcock et al. [47] reported a reduction in mortality risk of 19% in populations who have 30 minutes daily of moderate intensity activity 5 days per week, compared with those people with no activity. A longitudinal study among Scandinavian adults, for example, found that all-cause mortality rates in moderately and highly active persons decreased by 50% when compared to a sedentary group of people [53]. In addition, this study suggested that cycling to work would reduce the risks of all-cause mortality by approximately 40%. A study conducted in Copenhagen followed up a health cohort including 13,375 women and 17,265 men for nearly 15 years. The main finding of this study suggested that cycling to work can decrease the risk of all-cause mortality by 40%, including leisure time physical activity [53]. A similar result was reported in a Chinese cross-sectional study showing that women who regularly did physical exercise or used a bicycle as transportation could attain a 20–50% lower risk of premature mortality [54]. Further, Australian research revealed that a 5% increase in the proportion of people doing 30 minutes moderate activity each day could save around 600 lives per year, which could significantly reduce health expenditure to the health system [55].

*Active Transport, Physical Activity, and Benefits Relating to Specific Conditions*. Moderate intensity physical activities, including walking and cycling, have also been demonstrated to decrease the morbidity of many chronic diseases such as diabetes, cardiovascular disease, breast cancer, colon cancer, and dementia [11, 56–59]. Jeon and colleagues [57] reviewed 10 prospective cohort studies to estimate the effect of physical activity of moderate intensity on type II diabetes. They found that the risk of type II diabetes was 31% less for participants who engaged in regular moderate intensity physical activity, with 30% less risk among a regular walking population compared with almost no walking [57]. Xu et al. [60] systematically examined the relationship between active transport to work or school and cardiovascular health. A significantly positive association between active transport to work or school and cardiovascular health has been found in this review. Furthermore, another systematic review conducted by Monninkhof and colleagues reported that regular exercise might reduce the risk of postmenopausal breast cancer by 20%–80%, with each additional hour of physical activity per week potentially resulting in a further 6% reduction in breast cancer risk [58].

*Active Transport, Physical Activity, and Benefits Relating to Fitness and Weight*. Body fitness can also be strengthened by prompting active transport. A British study compared the physical condition of children who walked or cycled to school compared with those who travelled by bus or car. Their finding revealed that the former group was fitter than the latter one, with 30% higher vigour in boys who took active transport and seven times higher in girls [61]. It is estimated that approximately 40 million children and 1.4 billion adults are either overweight or obese worldwide [62]. Active travelling may be regarded as an efficient approach to combat obesity. Indeed, a synthesised result from the systematic review conducted by Xu et al. also reported that more active transport to work or school has been found associated with lower body weight [60]. Moreover, an Australian study conducted by Ming Wen and Rissel suggested that men who drove to work were more likely to be obese or overweight compared with those who chose cycling [49]. Recent research investigating the obesity levels in Europe, North America, and Australia established an inverse relationship between active transport levels and obesity levels in the population [63]. The results suggested that active transport might be one of the important factors contributing to international differences in obesity rates [63].

### 3.3. Economic Cobenefits

In addition to environmental and health benefits, economic benefits can also be obtained through alternative transport promotion. At the moment, the majority of motorised vehicles are highly dependent on fossil oil and consume almost 50% of total fossil oil usage [64]. The overreliance of motorised vehicles on petroleum not only causes concern regarding GHG emissions but also leads to nonrenewable energy sources diminishing. Apparently, the fossil oil cost would be reduced with the reductions of vehicle kilometres travelled and the increase of alternative transport [65]. In addition, with the decrease in the vehicle travelled kilometres and fuel use, the costs of air pollution control would also be reduced correspondingly. Although the costs per kilometre air pollution and climate change of public transport are higher when compared with private transport, the costs per passenger of public transport are significantly less than that of private transport due to the large number of private cars [66].

A New Zealand case study estimated the total costs of private and public transport in Auckland, with particulate matter as the vehicle-related air pollution indicator. Their finding demonstrated that a PM_10_ value of 16 mg/m^3^ caused by motor vehicle exhaust led to additional illnesses and amounted to a cost of $422 million in 2001, which was equal to 57% of the total health cost arising from PM_10_ in the Auckland region [66]. Further analysis on these additional costs revealed that $211.6 million (of the $422 million) came from private transport, whereas only $17.2 million was contributed by public transport. A similar trend was also observed when the authors calculated the total climate change costs from private cars and public transport using a unit cost per tonne CO_2_. By this standard, the total costs from transport were $58.4 million, with $0.67 million coming from public transport and $57.8 million from private transport [66]. In addition to the cost reduction in fossil oil usage and air pollution control, economic benefits of alternative transportation may also be achieved by reducing motor vehicle-related mortality and morbidity. In Australia, it has been reported that the combined economic cost of motor vehicle-related mortality and morbidity was approximately $2.7 billion in 2000. More than 85% of this cost was incurred in capital cities, which covered 80% of Australian populations [67].

Previous studies have also suggested that negative health outcomes caused by physical inactivity (related to car travel, rather than alternative transport) might lead to increased medical expenditure as well. Recent estimates indicate that the direct and indirect costs are $13.8 billion for physical inactivity [55] and $21 billion for obesity and overweight in Australian [68]. To investigate the economic benefit from active transport, Grabow and Colleagues [14] modelled the impact on the health budget of eliminating short motor vehicle trips in 11 metropolitan areas in the upper mid-western United States. They estimated that the combined benefits of improved air quality and physical fitness would exceed $8 billion/year.

## 4. Methodology Issues in Cobenefit Analysis

### 4.1. Scenarios

To predict the cobenefits of alternative transportation modes, it is fundamental to set up alternative GHGs emission/active transportation scenarios for analysis. Alternative scenarios are designed not only based on the researcher's assumptions but also in relation to local transport circumstances. Although there are a variety of alternative transport choices, such as bus, taxi, hybrid vehicle, and bicycle, most researchers tend to choose ecofriendly and healthy modes to evaluate the cobenefit effects. In current cobenefit studies (as seen in Table 1), the alternative transport scenarios were built from different perspectives, and different assumptions were made with a consideration of uncertainties. However, those assumptions must be reliable, practical, and achievable. For instance, in a US study [14], all short car trips (≤8 km) were assumed to be eliminated and they made this scenario based on a census-tract level travel. Similarly, Woodcock et al. [11] compared a BAU with alternative scenarios in 2030 in London, UK, and Delhi, India, for their cobenefit analysis. In their study, because of the different traffic structure, they then modelled different alternative transport scenarios for each city. Obviously, it is not practical to assume that 100% of car trips will be made by cycling or public transport. It would be preferable, instead, to base scenarios on an understanding of local traffic conditions and future transport plans or policies of local authorities, although the challenges in doing this are acknowledged.

### 4.2. Modelling Method and Tool

#### 4.2.1. Environmental Benefit Assessment

In transport related cobenefit studies, the estimation of the emissions change from motor vehicle reduction is a vital component of the environmental impact assessment. There are various vehicle emission models which could be used in cobenefit analysis appropriately. However, data requirements and modelling approaches may vary for each model, and it is difficult to judge which one is the best. Generally, the ideal model should be adapted to the target application and the changing demand. In addition, the model should be used either to examine relative changes from different scenarios or to predict absolute levels of emissions under a given period and location [69]. Furthermore, it is also vital for researchers to model emissions with tools corresponding to local traffic situations. For example, a UK study [11] modelled the vehicle emissions in London by using the Emissions Toolkit developed by the Environmental Research Group from King's College, which provided detailed transport emission data for over 6,000 roads in London. In a New Zealand study [70], the researchers used the Vehicle Emissions Prediction Model (VEPM), which was developed by Auckland Regional Council as their own emission model, to calculate the average light vehicle emissions [10]. Rojas-Rueda et al. [13] and Grabow et al. [14] also used local air pollution model to assess the health impacts of changing in PM concentrations in Barcelona and midwestern United States under the car trips reduction scenario. PM is a complex mixture of extremely small particles and liquid droplets. It can be of organic or inorganic origin and includes airborne dust particles, soot and hydrocarbons from combustion processes, metal residues, fibres, and sulphate or nitrate compounds [71]. Meanwhile, there have been much strong lines of evidence, relating to proving the dose-response relationship between PM and health outcomes [44, 72–74]. Thus, to avoid double counting, the WHO suggests using PM_10_ and PM_2.5_ as the indicators of air pollution exposure. All the cobenefit studies we reviewed in this review chose PM_10_ or PM_2.5_ concentration as the major air pollution exposure indicator in their health impact assessment section. 

Other general tools could be used to estimate the change in emissions if there is a lack of local air pollution modelling tools. A new generation of emission models, including The Vehicle Air Pollution Information System (VAPIS) model and The Simple Interactive Models for better air quality (SIM-air), have been developed recently [75] as user friendly spreadsheet based tools. One of the advantages of these models is that only basic local traffic parameters are required, such as baseline vehicular numbers, average annual vehicle growth rates, average vehicle travelled kilometres, and emission factors. Therefore, these models can still be applied when available data are limited. For example, the SIM-air Model was used to estimate PM_2.5_ for Delhi where local traffic data were incomplete in the UK study [11]. Although the emission trends analysis can be performed easily, the estimations generated from these tools are relatively crude. It is also impossible for the SIM-air Model to assess the impact on emissions induced by traffic-management schemes, such as speed restriction, roundabouts, signal coordination, or road widening. Despite the limitations, however, these tools still remain a substitute when detailed local traffic information is not available for cobenefit analyses.

In cobenefit studies, the selection of an air pollutant index related to vehicles should have a close association with population health impact. Although air pollutants are various, it is not necessary to model all the vehicle pollutant emissions since air pollution-related diseases are often caused by one or two dominated pollutants [76]. PM is a complex mixture of extremely small particles and liquid droplets. It can be of organic or inorganic origin and includes airborne dust particles, soot and hydrocarbons from combustion processes, metal residues, fibres, and sulphate or nitrate compounds [71]. Moreover, there have been much strong lines of evidence, relating to proving the dose-response relationship between PM and health outcomes [44, 72–74]. Thus, to avoid double counting, the WHO suggests using PM_10_ and PM_2.5_ as the indicators of air pollution exposure. All the cobenefit studies we reviewed in this review chose PM_10_ or PM_2.5_ concentration as the major air pollution exposure indicator in their health impact assessment section. 

#### 4.2.2. Health Benefit Assessment

Health benefit assessment is another critical element in cobenefit studies. A scoping method was recently developed by the IPCC and WHO, in conjunction with other international organizations, to estimate the health impact from greenhouse mitigation strategies [77]. This method was then modified slightly by Smith and Haigler to remain consistent within energy cobenefit studies [78]. The development of these scoping methods has made it possible to extend cobenefits analyses to more sophisticated assessments. In the scoping methods, Comparative Risk Assessment (CRA) plays an essential role in evaluating the health benefits of interventions in the energy sector. Developed by WHO, CRA is defined as the systematic evaluation of the changes in population health which result from modifying the population distribution of exposure to a risk factor or a group of risk factors [79]. In simple terms, this parameter could be used to evaluate the change of attributable fractions (AF) of risk factors and translate the changed AF into burdens of disease which can be applied to the projection by the researchers. Therefore, this approach cannot only be used to assess the health benefits from enhanced physical activity by increasing active transport but also can be adapted to evaluate the change in disease burden of air pollution reduction. As we can see from Table 1, both the UK study [11] and the Bay Area study [15] used this approach to comprehensively estimate health impacts of alternative transport scenarios. In addition, the CRA has been used to evaluate the health cobenefits of many mitigation activities for GHG emissions and air pollution [11, 80–82]. However, it is notable that assumptions and values of key parameters have an important effect on the model outcomes. For example, it is necessary to obtain the values of RR of risk factor like air pollution or physical inactivity. If there is no local statistical data available, the *RR* could be estimated by doing meta-analysis like the UK [11], and Bay Area studies [15]. As seen in Table 1, other studies used an indirect approach with the Health Economic Assessment Tool (HEAT) to evaluate the health impact. We will discuss this tool in the following section.

The respiratory and cardiovascular systems appear to be the most affected by urban air pollution. The WHO estimates the disease burden of air pollution based on the contributions of three health outcomes: mortality from cardiopulmonary disease in adults, mortality from lung cancer, and mortality from acute respiratory infections (ARI) in children aged 0–4 [83]. According to the global burden of disease calculated by the WHO, diseases having the most significant association with physical inactivity include diabetes, dementia, hypertensive heart disease, ischemic heart disease, cerebrovascular disease, breast cancer, colorectal cancer, and depression. Therefore, as shown in Table 1, most of the studies reviewed conducted the health impact assessment on air pollution reduction based on the cardiopulmonary disease and lung cancer health outcomes, and the health impact assessment on active transport based on major chronic diseases outcomes. Health outcomes could be considered in terms of the number of deaths, mortality and morbidity rates, and the Disability Adjusted Life Years (DALY). DALY is a metric that combines premature mortality and morbidity, which can provide an overall picture of burden of disease. 

One issue in health benefit assessment is that enhanced physical activity improves health effects gradually over time and those effects will be maintained in a certain time period. Therefore, how quickly the health benefit of increasing an individual's active transport level appears remains uncertain. As such, current cobenefit studies only projected the health cobenefits that occurred in one “accounting year.”

Another controversial issue concerns whether active transport is associated with more physical activity in reality. One systemic review conducted by Faulkner et al. suggested that children and youth who actively travel to school tend to be more physically active than passive commuters [48]. However, another recent systematic review concluded that evidence that active transport users necessarily have more physical activity than others is limited due to a lack of longitudinal studies [84]. Therefore, it remains uncertain whether active transport commuters gain health benefits solely from their active transport use. 

Furthermore, the UK and Bay Area studies [11, 15] assumed everyone in the population aged over 15 would possibly use active transport as an alternative; a goal very hard to achieve because older people constitute a group that may find it difficult to start cycling for various reasons. In Denmark, cycling trips declined with age, but even among 70–74 years old people, cycling still accounts for 12% of all their trips, double that percentage has been found in Dutch elderly [27]. Thus, it is worth considering whether elder people should be included in the health impact model. On the other hand, considering those people already achieved the criteria of sufficient physical activity, they may not gain more health benefit from active transport since physical inactivity was no longer a risk factor of some diseases for them. Accordingly, largest health benefits from active transport may be gained from those people who are completely sedentary but become active travellers.

### 4.3. Economic Benefit Assessment

Economic benefit assessment in a cobenefits study can be conducted from different perspectives, such as investment cost on environmental protection, fuel savings, and cost of medical expenditure. A standard “value of a statistical life” approach is commonly used in transport appraisals, which reflects the willingness of a middle-aged person to pay to avoid sudden death (willingness-to-pay) [85]. The value of willingness-to-pay could vary considerably between different regions. Alternatively, Smith and Haigler [78] recommended a simpler way to assess the cost effectiveness of possible interventions by comparing local gross domestic product (GDP) with DALYs. If the health-related investment is less than local $GDP/capita per DALY, the intervention is considered to be very cost effective and should be promoted quickly and widely [78]. The intervention is cost-effective if the health-related investment is between one and three times of the local $GDP/capita per DALY. When an investment is over three times of the local $GDP/capita per DALY, the intervention is not considered to be cost effective.

WHO has developed a specific Health Economic Assessment Tool (HEAT) to evaluate the health effects related to increased cycling [86]. This Excel-based tool sets the relative risk as 0.72 for all-cause mortality of regular adult commuter cyclists. The HEAT also contains a default value of a statistical life, based on the Copenhagen Centre for Prospective Population studies which controlled gender, smoking, education, leisure time physical activity, body mass index, and other risk factors for chronic disease [53]. Therefore, reduced mortality could be used as an indicator to estimate the mean annual benefit from cycling. The total value of economic savings due to the reductions in all-cause mortality among these cyclists could also be calculated with the data entered by the user. Those studies [12–14] that conducted the health impact assessment by the HEAT have not considered the age issue of beneficiaries, because HEAT is only designed for adult population (aged approximately 20–64 years) and it is generally accepted that this group of people is most suitable for cycling. However, the HEAT is not suitable for assessing the economic benefits of other alternative transport, such as walking, as the Copenhagen study only compared the relative risk of all-cause mortality between cyclists and noncyclists. In addition, the HEAT only investigates the impact on mortality but not on morbidity and it does not consider mental health issues. It also cannot be applied to children. 

### 4.4. Data Issues

One of the challenges in conducting a cobenefit study is the establishment of a series of modelling work to project the multiple benefits. To establish effective modelling relies on multiple high quality datasets. For instance, to estimate environmental benefit, it is essential to collect transport data, such as annual vehicle kilometres travelled, emission factors of vehicle types, and public travel patterns. For health benefit estimations, various health data are needed, such as prevalence of insufficient physical activity, local mortality and morbidity of relevant diseases, and relative risks of air pollution and physical inactivity. When projecting long-term effects of alternative transport plans, baseline data quality is crucial. To date, data between different countries have shown heterogeneity. In addition, transferability between diverse populations has not been established yet. In addition, transferability between diverse populations is challenging for researchers, who need to consider the comparability and differences between populations and regions. Theoretically, it is ideal to use local data as the baseline when calculating the estimations. However, the available local databases of transport, emissions, and health system are updated in different years, and it would be acceptable to use databases in different years to build the baseline scenario. For studies which tend to make projective model, researchers also need to consider the development trend in the study population. Like the UK and Bay Area studies [11, 15], they took into account population growth and changes in emission standards when they designed the 2030 scenarios. Moreover, vehicular emission factor, a key parameter for air pollution modelling, varies with ageing vehicle fleets, engine types, or on the cold start/driving/brake, which should be considered in order to adjust the model. 

### 4.5. Summary and Recommendations

It is well known that alternative transportation can bring cost-effective benefits to environmental protection and population health. However, current analyses of transport mitigation strategies in terms of health and economic aspects are still at an early stage. Most of the previous research regarding the transport sector has only focused on one of those possible benefits and has rarely quantified the overall cobenefits of alternative transportation planning. Additionally, most of the current cobenefit studies are more interested in the long-term effect of alternative transportation, while the short-term effect has not been considered adequately. Some other benefits, such as social benefits from alternative transport are also valuable for further investigation. For example, both cycling and walking could enhance social/neighbourhood interaction. It has been shown that active transport can increase activity in local neighbourhoods and the passive surveillance of private and community infrastructure [87].

The health effects of active transport could involve physical, mental, and psychological aspects. At the moment, studies thoroughly investigating all these aspects are relatively rare. The majority of the conclusions from existing cobenefit studies only indicate that active transport has positive influences on preventing chronic disease. It is still uncertain how much mental and psychological health benefit could be achieved by the alternative transport. Moreover, the benefits of noise mitigation have also been commonly neglected, which is a particular effect induced by motor vehicle reduction. There are also some gaps in health benefit research when taking different age groups into consideration. Although researchers could assume that people of all the ages would be affected by air pollution reduction and increased physical activity due to reduced usage of motor vehicles, such hypotheses may lead to inaccurate conclusions for certain population, such as children, for which few studies have considered the health benefits.

At the moment, the investigation of economic benefits of transport strategies, especially for active transport, is still at an early stage. The assessment tools currently available for economic benefits analyses can be limited when applied to different scenarios. For example, WHO has provided HEAT, which is a specific economic assessment tool for cycling. However, how to apply it to other active transport modes (such as walking) still needs to be explored. The range of current economic benefit assessment is also somewhat incomplete. Fuel saving, decreasing investment in environmental protection, and medical expenditure reduction are the aspects which have been most commonly studied. Beyond these, other economic benefits related to reducing traffic congestion, such as car space requirements and oil demands, should also be taken into account in the future.

To date, most of the cobenefits research has been conducted in developed societies. Relevant studies in under-developed societies are insufficient, especially in countries such as China and India where motor vehicle emissions have become a significant source of air pollution due to the recent sharp increase in vehicle numbers. As different types of cities have dissimilar populations, traffic situations, transportation modes, weather types, and infrastructures, cobenefit studies specific to individual regions are essential, and further research should be applied to cities with different characters. Furthermore, as well as considering alternative transport, cobenefits of mitigation strategies in other energy consuming sectors, such as industry, agriculture, and electricity generation, are also significant and warrant further exploration.

In addition to study design, choice of methods may also cause bias in the research of cobenefits. While quantitative methods are the most frequently used, qualitative methods have been rarely used in studying cobenefits. Although public behaviour and stakeholder attitudes may influence transport choice and policy making, little information has been provided in this area, which is well suited to in-depth qualitative analysis. Thus, further investigation should adopt qualitative methods, such as interviews and focus group discussions, in order to address these gaps in knowledge. Findings from the combination of both quantitative and qualitative methods will provide stronger evidence to assist policy makers in decision making and policy implementation.

## Figures and Tables

**Table 1:** Summary of cobenefits studies in transport area.

Reference	Study design	Methodological of modeling									Results
Author, year, study sites	Scenarios	Environment impact assessment			Health impact assessment			Economic impact assessment			Main finding
		Method/tools	Indicators	Key parameters resource	Method/tools	Indicators	Key parameters resource	Method/tools	Indicators	Key parameters resource	
Woodcock et al. (2009) [11] London, UK, and Delhi, India	BAU 2030 Lower-carbon-emission motor vehicles (more efficient engines and fuels switching) Increased active travel (increasing walking and cycling) Towards sustainable transport (lower-carbon-emission motor vehicles and increased active transport scenarios)	London: ERG Emissions Toolkit ADMS 4 OSPM v5.0.64 Delhi: SIM-air Version 1.3	Annual mean PM_10_ and PM_2.5_ concentrations	London: LAEI 2006 Delhi: inventory of aerosol and sulphur dioxide emissions from India	CRA	Annual preventable DALYs of cardio-respiratory lung cancer,acute respiratory infections (air pollution reduction), diabetes, dementia, hypertensive heart disease, cerebrovascular disease, breast cancer, colorectal cancer, depression (increased physical activity)	Global Burden of Disease Database Meta-analyses from an exhaustive literature review London: London-area travel demand models London Area Travel Survey Delhi: World Health Survey 2000				London: 60% reduction in transport CO_2_ emissions from the 1990 levels; 7439 DALYs per million population would be avoided (towards sustainable transport scenarios) Delhi: 199% increase in CO_2_ emissions from 1990; 12 995 DALYs per million population would be avoided (towards sustainable transport scenarios)

Lindsay et al. (2011) [12] Auckland, New Zealand	Moving short urban trips (<7 km) from cars to bicycles by 1%, 5%, 10%, and 30%	VEPM version 2.3	vehicle emissions per km for CO, CO_2_, NO_*X*_, VOC, and PM_10 _	HAPiNZ study	HEAT	Annual reduction in deaths Energy expenditure	New Zealand Household Travel Survey HAPiNZ study	HEAT	Value of Statistical Life Fuel savings ($NZ)	HAPiNZ study Ministry of Transport's Value of Statistical Life	Shifting 5% of vehicle kilometers to cycling would save 22 million liters of fuel; reduce transport- related GHGs by 0.4%; avoide 122 deaths annually due to increased physical activity and local air pollution reduction; save $200 million per year from health effect

Rojas-Rueda et al. (2012) [13] Greater Barcelona metropolitan area	BAU Replacing car trips (20%, 40%) by bicycle Replacing car trips (20%, 40%) by public transport (bus, tram, train, and metro) Replacing car trips (20%, 40%) by bicycle and public transport	Barcelona Air-Dispersion Model	PM_2.5_ concentration CO_2_ emission	Barcelona City council report 2009 Local transportation departments	RR functions in PM2.5 HEAT	All-cause mortality	Daily Mobility Survey of Catalonia Daily Mobility Survey of Catalonia Statistical Institute of Catalonia Published literature				A shifting of 40% car trips to cycling and public transport would avoid 98.5 deaths in total; reduce 203, 251 t/CO_2_ emissions per year

Grabow et al. (2012) [14] Midwestern United States	Replacing short car trips (≤8 km round trip) in urban areas by bicycle	Community Multiscale Air Quality Model version 4.6 BenMAP version 4.0.35	Mean annual PM_2.5_ and O_3_ concentration	2001 National Emissions Inventory US EPA	BenMAP HEAT	Mortalities of asthma, chronic bronchitis, respiratory problems, cardiovascular problems, work-loss days, acute respiratory symptoms, ER visits, mortality, HA (respiratory school-loss days worker productivity), (air pollution reduction), All-cause mortality (increased physical activity)	1996 National Health Interview Survey Published literature US EPA	BenMAP HEAT	Cost savings	US EPA	Eliminating short car trips and completing 50% of them by bicycle would decline mean annual PM_2.5_ by 0.1 *μ*g/m^3^;decline mortality by 1,295 deaths/year in 31.3 million people because of improved air quality and increased exercise; combine benefits of improved air quality and physical fitness would exceed $8 billion/year

Maizlish et al. (2013) [15] San Francisco Bay Area	BAU 2035 Low-carbon driving (increased hybrid vehicles and light-duty diesel, biofuel, and electric vehicles) Active transport (50% of BAU miles travelled in car trips less than 1.5 miles are walked and 50% of BAU miles travelled in car trips 1.5 to 5 miles are by bicycle)	EMFAC2007 BAAQMD air shed model	CO_2_ emissions Annual PM2.5 concentration	California Air Resources Board Bay Area Air Quality Management District	CRA	Lung cancer, respiratory disease, (air pollution reduction) Annual preventable DALYs of cardiovascular diseases, colon cancer, breast cancer, diabetes, dementia (increased physical activity)	Global Burden of Disease Database 2000 Bay Area Travel Survey Meta-analyses				Increasing active transport scenario would reduce 5952 DALYs per million people in total; increase the traffic injury burden by 39% (5907 DALYs); decrease GHGE by 14% Low-carbon driving scenario would reduce 31 DALYs per million people in total; reduce GHGE by 33.5%

BAU: business as usual.

ERG: Environmental Research Group.

ADMS: Atmospheric Dispersion Modelling System.

OSPM: operational Street Pollution Model.

SIM-air: Simple Interactive Models for better air quality.

LAEI: The London Atmospheric Emissions Inventory.

DALY: The disability-adjusted life year.

VEPM: Vehicle Emissions Prediction Model.

HAPiNZ: Application of Health and Pollution in New Zealand.

HEAT: Health Economic Assessment Tool.

BenMAP: Environmental Benefits Mapping and Analysis Program.

EPA: Environmental Protection Agency.

EMFAC: Emission Factors model.

BAAQMD: Bay Area Air Quality Management District.

## References

[1] Priddle R (2002). *World Energy Outlook 2002*.

[2] Fulton L (2004). Reducing oil consumption in transport: combining three approaches.

[3] Kahn Ribeiro S, Kobayashi S, Beuthe M (2007). Transport and its infrastructure. *Climate Change*.

[4] Huo H, Zhang Q, He K (2011). Modeling vehicle emissions in different types of Chinese cities: importance of vehicle fleet and local features. *Environmental Pollution*.

[5] ETC/ACC (2005). *Air Emissions Spreadsheet for Indicators 2004*.

[6] Cao J, Emadi A (2012). A new battery/ultracapacitor hybrid energy storage system for electric, hybrid, and plug-in hybrid electric vehicles. *IEEE Transactions on Power Electronics*.

[7] Tanaka K, Berntsen T, Fuglestvedt JS, Rypdal K (2012). Climate effects of emission standards: the case for gasoline and diesel cars. *Environmental Science and Technology*.

[8] Sayeg P, Bray D (2012). Estimating changes in emissions from bus rapid transit: making best use of transport sector experience. *Wiley Interdisciplinary Reviews*.

[9] Buehler R, Pucher J, Buehler R, Pucher J (2012). International overview: cycling trends in Western Europe, North America, and Australia. *City Cycling*.

[10] OEH BioBanking glossary. http://www.environment.nsw.gov.au/biobanking/glossary.htm.

[11] Woodcock J, Edwards P, Tonne C (2009). Public health benefits of strategies to reduce greenhouse-gas emissions: urban land transport. *The Lancet*.

[12] Lindsay G, Macmillan A, Woodward A (2011). Moving urban trips from cars to bicycles: impact on health and emissions. *Australian and New Zealand Journal of Public Health*.

[13] Rojas-Rueda D, de Nazelle A, Teixido O, Nieuwenhuijsen MJ (2012). Replacing car trips by increasing bike and public transport in the greater Barcelona metropolitan area: a health impact assessment study. *Environment International*.

[14] Grabow ML, Spak SN, Holloway T, Brian S, Mednick AC, Patz JA (2012). Air quality and exercise-related health benefits from reduced car travel in the midwestern United States. *Environmental Health Perspectives*.

[15] Maizlish N, Woodcock J, Co S, Ostro B, Fanai A, Fairley D (2013). Health cobenefits and transportation-related reductions in greenhouse gas emissions in the San Francisco Bay area. *American Journal of Public Health*.

[16] Hickman R, Ashiru O, Banister D (2010). Transport and climate change: simulating the options for carbon reduction in London. *Transport Policy*.

[17] Parikesit D, Susantono B (2013). Strengthening the role of public transport. *Transport Development in Asian Megacities*.

[18] Bhattacharjee G, Bhattacharya S, Neogi S, Das SK (2010). CNG cylinder burst in a bus during gas filling—lesson learned. *Safety Science*.

[19] Besser LM, Dannenberg AL (2005). Walking to public transit: steps to help meet physical activity recommendations. *American Journal of Preventive Medicine*.

[20] Rissel C, Curac N, Greenaway M, Bauman A (2012). Physical activity associated with public transport use—a review and modelling of potential benefits. *International Journal of Environmental Research and Public Health*.

[21] Jun M-J (2012). Redistributive effects of bus rapid transit (BRT) on development patterns and property values in Seoul, Korea. *Transport Policy*.

[22] Panero M, Shin HS, Zedrin A, Zimmerman S (2012). Peer-to-peer information exchange on Bus Rapid Transit (BRT) and Bus Priority Best Practices.

[23] Poole RW, Rubin TA, Swenson C (2012). *Increasing Mobility in Southeast Florida: A New Approach Based on Pricing and Bus Rapid Transit*.

[24] Mees P, Dodson J (2011). *Public Transport Network Planning in Australia: Assesing Current Practice in Australia's Five largest cities*.

[25] Avery L (2011). *National Travel Survey: 2010*.

[26] Bureau of Transport Statistics (2012). 2010/11 household travel survey summary report.

[27] Pucher J, Buehler R (2008). Making cycling irresistible: lessons from the Netherlands, Denmark and Germany. *Transport Reviews*.

[28] UK Department for Transport (2004). *Walking and Cycling: An Action Plan*.

[29] South Australian Government (2002). *Adelaide Travel Patterns: An Overview*.

[30] Australian Local Government Association (2010). *An Australia Vision for Active Transport*.

[31] Martine G, Marshall A (2007). *State of World Population 2007: Unleashing the Potential of Urban Growth*.

[32] Wang T, Xie S (2009). Assessment of traffic-related air pollution in the urban streets before and during the 2008 Beijing Olympic Games traffic control period. *Atmospheric Environment*.

[33] Wang Y, McElroy MB, Boersma KF, Eskes HJ, Veefkind JP (2007). Traffic restrictions associated with the Sino-African summit: reductions of NOx detected from space. *Geophysical Research Letters*.

[34] Nitschke M, Tucker GR, Hansen AL, Williams S, Zhang Y, Bi P (2011). Impact of two recent extreme heat episodes on morbidity and mortality in Adelaide, South Australia: a case-series analysis. *Environmental Health*.

[35] Gamble JL, Hurley BJ, Schultz PA, Jaglom WS, Krishnan N, Harris M (2013). Climate change and older Americans: state of the science. *Environmental Health Perspectives*.

[36] Bai L, Morton LC, Liu Q (2013). Climate change and mosquito-borne diseases in China: a review. *Globalization and Health*.

[37] WHO Air quality and health. http://www.who.int/mediacentre/factsheets/fs313/en/index.html.

[38] Gordon M, Staebler RM, Liggio J (2012). Measured and modeled variation in pollutant concentration near roadways. *Atmospheric Environment*.

[39] Van Poppel M, Int Panis L, Govarts E, Van Houtte J, Maenhaut W (2012). A comparative study of traffic related air pollution next to a motorway and a motorway flyover. *Atmospheric Environment*.

[40] Gan WQ, Tamburic L, Davies HW, Demers PA, Koehoorn M, Brauer M (2010). Changes in residential proximity to road traffic and the risk of death from coronary heart disease. *Epidemiology*.

[41] Tsai D-H, Wang J-L, Chuang K-J, Chan C-C (2010). Traffic-related air pollution and cardiovascular mortality in central Taiwan. *Science of the Total Environment*.

[42] Gan WQ, Davies HW, Koehoorn M, Brauer M (2012). Association of long-term exposure to community noise and traffic-related air pollution with coronary heart disease mortality. *American Journal of Epidemiology*.

[43] Beelen R, Hoek G, van den Brandt PA (2008). Long-term effects of traffic-related air pollution on mortality in a Dutch cohort (NLCS-AIR study). *Environmental Health Perspectives*.

[44] Gan WQ, Koehoorn M, Davies HW, Demers PA, Tamburic L, Brauer M (2011). Long-term exposure to traffic-related air pollution and the risk of coronary heart disease hospitalization and mortality. *Environmental Health Perspectives*.

[45] WHO Global Recommendations on Physical activity for Health. http://www.who.int/dietphysicalactivity/factsheet_recommendations/en/index.html.

[46] Begg S, Vos T, Barker B, Stevenson C, Stanley L, Lopez A (2007). *Burden of Disease and Injury in Australia, 2003*.

[47] Woodcock J, Franco OH, Orsini N, Roberts I (2011). Non-vigorous physical activity and all-cause mortality: systematic review and meta-analysis of cohort studies. *International Journal of Epidemiology*.

[48] Faulkner GEJ, Buliung RN, Flora PK, Fusco C (2009). Active school transport, physical activity levels and body weight of children and youth: a systematic review. *Preventive Medicine*.

[49] Ming Wen L, Rissel C (2008). Inverse associations between cycling to work, public transport, and overweight and obesity: findings from a population based study in Australia. *Preventive Medicine*.

[50] Thompson PD, Buchner D, Pina IL (2003). Exercise and physical activity in the prevention and treatment of atherosclerotic cardiovascular disease: a statement from the Council on Clinical Cardiology. *Arteriosclerosis, Thrombosis, and Vascular Biology*.

[51] Knut V, Stefan F, Farideh R, Harald M (2011). Cycling and walking for transport: estimating net health effects from comparison of different transport mode users' self-reported physical activity. *Health Economics Review*.

[52] Oja P, Titze S, Bauman A (2011). Health benefits of cycling: a systematic review. *Scandinavian Journal of Medicine and Science in Sports*.

[53] Bo Andersen L, Schnohr P, Schroll M, Ole Hein H (2000). All-cause mortality associated with physical activity during leisure time, work, sports, and cycling to work. *Archives of Internal Medicine*.

[54] Matthews CE, Jurj AL, Shu X (2007). Influence of exercise, walking, cycling, and overall nonexercise physical activity on mortality in Chinese women. *American Journal of Epidemiology*.

[55] Stephenson J, Bauman A, Armstrong T (2000). The cost of illness attributable to physical inactivity in Australia: a report prepared for the commonwealth department of health and aged care and the Australian sports commission.

[56] Hamer M, Chida Y (2009). Physical activity and risk of neurodegenerative disease: a systematic review of prospective evidence. *Psychological Medicine*.

[57] Jeon CY, Lokken RP, Hu FB, Van Dam RM (2007). Physical activity of moderate intensity and risk of type 2 diabetes: a systematic review. *Diabetes Care*.

[58] Monninkhof EM, Elias SG, Vlems FA (2007). Physical activity and breast cancer: a systematic review. *Epidemiology*.

[59] Oguma Y, Shinoda-Tagawa T (2004). Physical activity decreases cardiovascular disease risk in women: review and meta-analysis. *American Journal of Preventive Medicine*.

[60] Xu H, Wen LM, Rissel C (2013). The relationships between active transport to work or school and cardiovascular health or body weight a systematic review. *Asia-Pacific Journal of Public Health*.

[61] Voss C, Sandercock G (2010). Aerobic fitness and mode of travel to school in english schoolchildren. *Medicine and Science in Sports and Exercise*.

[62] WHO Obesity and overweight. http://www.who.int/mediacentre/factsheets/fs311/en/.

[63] Bassett DR, Pucher J, Buehler R, Thompson DL, Crouter SE (2008). Walking, cycling, and obesity rates in Europe, North America and Australia. *Journal of Physical Activity and Health*.

[64] Woodcock J, Banister D, Edwards P, Prentice AM, Roberts I (2007). Energy and transport. *The Lancet*.

[65] Bollen J, van der Zwaan B, Brink C, Eerens H (2009). Local air pollution and global climate change: a combined cost-benefit analysis. *Resource and Energy Economics*.

[66] Jakob A, Craig JL, Fisher G (2006). Transport cost analysis: a case study of the total costs of private and public transport in Auckland. *Environmental Science and Policy*.

[67] Bureau of Transport and Regional Economics (2005). Health impacts of transport emissions in Australia: economic costs.

[68] Colagiuri S, Lee CMY, Colagiuri R (2010). The cost of overweight and obesity in Australia. *Medical Journal of Australia*.

[69] Wang H, Iain MG Review of vehicle emission modelling and the issues for New Zealand.

[70] Lindsay G, Macmillan A, Woodward A (2011). Moving urban trips from cars to bicycles: Impact on health and emissions. *Australian and New Zealand Journal of Public Health*.

[71] Government of South Australia Measuring Air Pollutants in Adelaide. Exceedances of NEPM Guidelines for Key Air Pollutants. http://www.denr.sa.gov.au/reporting/atmosphere/airqual/nepm.html#top.

[72] Dominici F, Peng RD, Bell ML (2006). Fine particulate air pollution and hospital admission for cardiovascular and respiratory diseases. *Journal of the American Medical Association*.

[73] Wong TW, Tam WS, Yu TS, Wong AHS (2002). Associations between daily mortalities from respiratory and cardiovascular diseases and air pollution in Hong Kong, China. *Occupational and Environmental Medicine*.

[74] Hertel O, Jensen SS, Hvidberg M (2008). Assessing the impacts of traffic air pollution on human exposure and health. *Road Pricing, the Economy and the Environment*.

[75] Guttikunda S urbanemissions.info. http://urbanemissions.info.

[76] Jalaludin B, Salkeld G, Morgan G, Beer T, Nisar YB (2009). A methodology for cost-benefit analysis of ambient air pollution health impacts.

[77] Zhao L, Xiao Y, Wang B, Xu X (2007). Linking climate policy with development strategy in Brazil, China, and India.

[78] Smith KR, Haigler E (2008). Co-benefits of climate mitigation and health protection in energy systems: scoping methods. *Annual Review of Public Health*.

[79] United States Environmental Protection Agency (2006). Greenhouse gas emissions from the US transportationm sector 1990–2003.

[80] Smith KR, Jerrett M, Anderson HR (2009). Public health benefits of strategies to reduce greenhouse-gas emissions: health implications of short-lived greenhouse pollutants. *The Lancet*.

[81] Markandya A, Armstrong BG, Hales S (2009). Public health benefits of strategies to reduce greenhouse-gas emissions: low-carbon electricity generation. *The Lancet*.

[82] Wilkinson P, Smith KR, Davies M (2009). Public health benefits of strategies to reduce greenhouse-gas emissions: household energy. *The Lancet*.

[83] WHO (2009). Global health risks: mortality and burden of disease attributable to selected major risks.

[84] Wanner M, Götschi T, Martin-Diener E, Kahlmeier S, Martin BW (2012). Active transport, physical activity, and body weight in adults a systematic review. *American Journal of Preventive Medicine*.

[85] Cavill N, Kahlmeier S, Rutter H, Racioppi F, Oja P (2008). Methodological guidance on the economic appraisal of health effects related to walking and cycling.

[86] Rutter H, Cavill N, Kahlmeier S, Dinsdale H, Racioppi F, Oja P (2007). *Health Economic Assessment Tool for Cycling (HEAT For Cycling)"*.

[87] Hume City Council (2010). *Walking and Cycling Strategy 2010–2015*.

